# Identification of 491 proteins in the tear fluid proteome reveals a large number of proteases and protease inhibitors

**DOI:** 10.1186/gb-2006-7-8-r72

**Published:** 2006-08-10

**Authors:** Gustavo A de Souza, Lyris MF Godoy, Matthias Mann

**Affiliations:** 1 Center for Experimental BioInformatics (CEBI), Department of Biochemistry and Molecular Biology, University of Southern Denmark, Campusvej, DK-5230 Odense M, Denmark; 2 Department of Proteomics and Signal Transduction, Max Planck Institute of Biochemistry, Am Klopferspitz, D-82152 Martinsried, Germany

## Abstract

**Background:**

The tear film is a thin layer of fluid that covers the ocular surface and is involved in lubrication and protection of the eye. Little is known about the protein composition of tear fluid but its deregulation is associated with disease states, such as diabetic dry eyes. This makes this body fluid an interesting candidate for in-depth proteomic analysis.

**Results:**

In this study, we employ state-of-the-art mass spectrometric identification, using both a hybrid linear ion trap-Fourier transform (LTQ-FT) and a linear ion trap-Orbitrap (LTQ-Orbitrap) mass spectrometer, and high confidence identification by two consecutive stages of peptide fragmentation (MS/MS/MS or MS^3^), to characterize the protein content of the tear fluid. Low microliter amounts of tear fluid samples were either pre-fractionated with one-dimensional SDS-PAGE and digested *in situ *with trypsin, or digested in solution. Five times more proteins were detected after gel electrophoresis compared to in solution digestion (320 versus 63 proteins). Ontology classification revealed that 64 of the identified proteins are proteases or protease inhibitors. Of these, only 24 have previously been described as components of the tear fluid. We also identified 18 anti-oxidant enzymes, which protect the eye from harmful consequences of its exposure to oxygen. Only two proteins with this activity have been previously described in the literature.

**Conclusion:**

Interplay between proteases and protease inhibitors, and between oxidative reactions, is an important feature of the ocular environment. Identification of a large set of proteins participating in these reactions may allow discovery of molecular markers of disease conditions of the eye.

## Abstract 2

A proteomic analysis of the tear fluid suggests that an interplay between proteases and protease inhibitors, and between oxidative reactions, is an important feature of the ocular environment.

## Background

The eye is covered by a thin, fluid film that serves several functions. It has critical roles in the optical system, lubricates the eye, provides nutrients and growth factors to the epithelium and serves as a barrier to the outside environment [1,2]. In the last function, it protects the eye against infection. The tear film is an aqueous layer containing proteins and electrolytes secreted by the lacrimal gland situated within the orbit above the lateral end of the eye, and other accessory gland secretions. Additionally, tear fluid is in contact with the epithelium of the lid and, thereby, is in indirect contact with the blood circulation. Major tear proteins include lysozyme, lactoferrin, secretory immunoglobin A, serum albumin, lipocalin and lipophilin [3]. The function of lysosyme, for example, is to lyse bacterial cell walls.

Tear fluid has become a body fluid of interest because it contains proteins in high concentration (about 8 μg/μl), it is relatively easy to collect, and several reports indicate that changes in its protein content can reflect normal or disease states. For example, electrophoretic and chromatographic analyses suggest that the tear protein patterns of diabetic patients are very different from those of healthy subjects [4,5]. Biochemical characterization of tear proteins is also important for understanding tear deficiencies, contact lens incompatibilities, tear film instabilities and several other eye diseases.

Qualitative and quantitative techniques that have been applied to the study of the tear proteome include one- and two-dimensional electrophoresis [6,7], enzyme-linked immunosorbent assay (ELISA) and high-performance liquid chromatography techniques [4]. More recently, analytical methods that couple microliter sample size with high sensitivity and resolution have been used in detailed studies of changes in tear composition following injury or disease. These methods have been used to map tear protein profiles, and include several mass spectrometry technologies, such as matrix assisted laser desorption ionization-time of flight (MALDI-TOF), surface-enhanced laser desorption ionization-TOF (SELDI-TOF) and liquid chromatography coupled with electrospray ionization (LC/MS) [8-11].

However, despite these efforts to identify and catalogue the proteins present in the tear, only a very limited number of proteins have been described in the literature. Patterns obtained in two-dimensional gel electrophoresis suggest that tear fluid contains at least 200 proteins [12] and an LC/MS study of intact proteins indicated at least 17 different molecular weights [8]. More recently, Li *et al*. [13] identified 54 different proteins using a combination of different proteomic approaches. Using a membrane-bound antibody array, Sack *et al*. [14] detected 80 different cytokines, chemokines and growth factors in tear samples. We were able to retrieve a total of about 60 described identifications and Harding [15] mentions a tear fluid proteome of about 80 proteins, including proteins only present in special conditions, such as allergy. The relatively low number of proteins identified, compared to other body fluids, may be due to the limited sensitivity of the methods employed [16], as well as the challenging composition of the tear fluid proteome, in which three proteins (lipocalin, lysozyme and lactoferrin) correspond to approximately 80% of the total protein concentration [17].

Recent developments in mass spectrometry-based proteomics (reviewed in Aebersold and Mann [18]) have dramatically increased our ability to analyze complex proteomes in-depth. In particular, a hybrid instrument, the linear ion trap-Fourier transform (LTQ-FT) mass spectrometer, combines very fast sequencing speed and high sensitivity with high resolution and mass accuracy [19]. We have recently described very high confidence protein identification by a combination of extremely accurate peptide mass measurement with two stages of peptide fragmentation [20]. These MS^3 ^spectra are scored with a probability based algorithm, which significantly adds to the confidence of peptide identification and allows 'rescue' of proteins identified with only one peptide. In our laboratory, this instrument has allowed the unambiguous identification of low abundant proteins in signaling pathways and organelles [21,22]. In addition, we also used the very recently developed LTQ-Orbitrap mass spectrometer [23] for analysis of tear fluid. In this instrument, ions are detected with high resolution by their motion in a spindle shaped electrode, instead of in a high magnetic field as is the case in the LTQ-FT spectrometer. We have recently shown that, by using a 'lock mass strategy', very high mass accuracy is routinely achievable in both the MS and MS/MS mode [24], which virtually eliminates the problem of false positive peptide identification in proteomics, and it is much easier than previously possible to identify post-translational modifications.

Here, we used both mass spectrometers in the analysis of the tear fluid proteome and report the unambiguous identification of 491 proteins. We observed a large number of proteases (32 proteins) and protease inhibitors (also 32 proteins), most not previously described as components of the tear fluid. In addition, we also identified 18 proteins that are involved in the anti-oxidant activity of the tear, of which 16 were not described previously. This in-depth analysis of the tear fluid should be of interest in ophthalmology and the results can be used as a reference to allow future characterization of disease states reflected in the tear fluid.

## Results

### Comparison between in-gel and in-solution digestion

**Figure 1:**
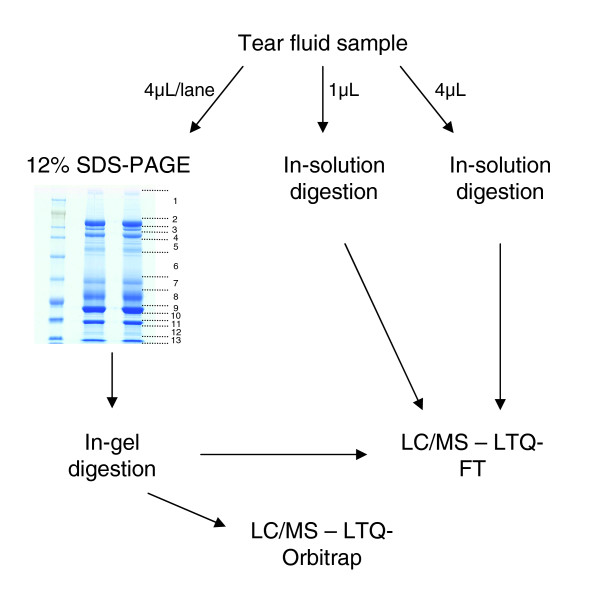
Approach used for tear fluid analysis. The tear fluid was analyzed by both in-solution digestion (1 and 4 μl) and one-dimensional gel separation combined with MS (GeLC-MS; 2 lanes of 4 μl each) on a LTQ-FT, and also through GeLC-MS on a LTQ-Orbitrap. The numbers indicate the bands according to the slicing pattern used for sample fractionation prior to *in situ *digestion.

To establish optimal conditions for determination of the tear fluid proteome we performed both in-gel and in-solution digestion, as summarized in Figure 1. Tear fluid was subjected to SDS-PAGE, the gel band cut into 13 slices, in-gel digested with trypsin and the resulting peptide mixtures analyzed by LC MS^3 ^in the LTQ-FT. Alternatively, proteins were digested in solution with Lys-C, or alternatively by Lys-C followed by trypsin, prior to MS analysis. Our results showed a total of 320 proteins identified for in gel-based analysis whereas only 59 proteins were identified using 1 μl of tear digested in solution and 63 proteins for 4 μL of in solution tear digestion.

**Figure 2:**
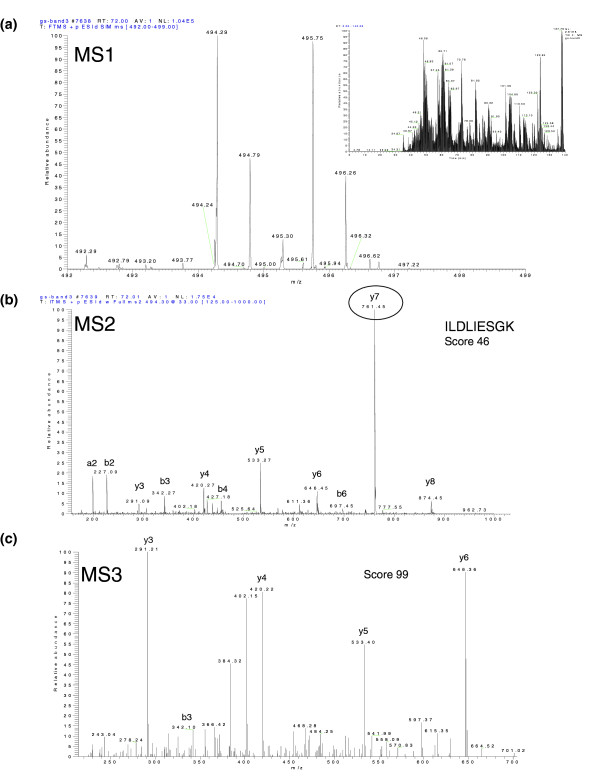
LTQ-FT data for the peptide at *m/z *494.29 (ILDLIESGK). The figure shows an example of data-dependent acquisition on the LTQ-FT. **(a) **SIM scan of the doubly charged peptide at 494.29, observed in the total ion chromatogram. **(b) **The peptide is selected for fragmentation and MS^2 ^acquisition, and **(c) **the most intense daughter-ion is selected for a new round of fragmentation MS^3^. Partial data obtained in the MS^3 ^is used to confirm sequence observed in the MS^2 ^and, consequently, improves the probability score for the identified sequence.

Figure 2 illustrates an example of MS acquisition and identification for in-gel digestion. In the inset of Figure 2a, the total ion chromatogram (TIC) is represented, and the spectrum shows the ions detected in selected ion monitoring (SIM) mode. Figure 2b shows the fragmentation pattern of the most intense ion in Figure 2a (m/z = 494.2906, peptide mass = 987.5812 Da). The identification is initially done on the basis of the data obtained using the Mascot algorithm [25]. Figure 2c shows the MS^3 ^of the most intense ion observed in Figure 2b, which is used to support or discard the identification made on the basis of MS^2 ^spectra [20,26].

### Comparison between LTQ-FT and LTQ-Orbitrap analysis

**Figure 3:**
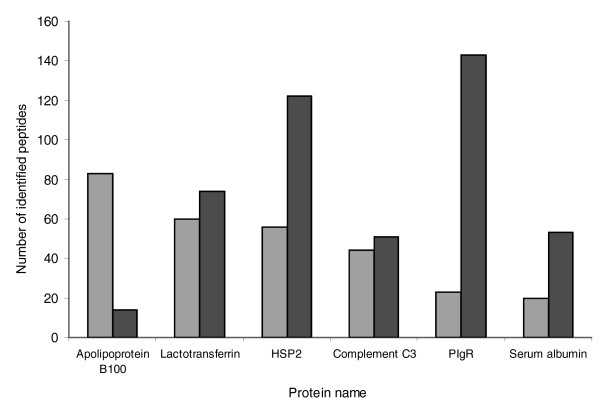
Data comparison between LTQ-FT and LTQ-Orbitrap spectrometry. The numbers of peptides for the top six identified proteins (LTQ-FT data) were compared between the two methods. Except for the protein Apolipoprotein B100, we observed a significant increase in the number of peptides identified with the LTQ-Orbitrap. This pattern was observed for most of the proteins identified with more then three peptides in the LTQ-FT. Light gray bars represent LTQ-FT data, dark gray represents LTQ-Orbitrap data.

As shown in Figure 1, *in situ *digestion of 4 μl of tear sample was also analyzed by the LTQ-Orbitrap mass spectrometer. In this case, we were able to identify 368 proteins in the sample. Since MS^3 ^analyses were not performed in the LTQ-Orbitrap, the criteria used for protein identification required at least two peptides with statistical significance (see Materials and methods). When the LTQ-FT protein list was overlaid with the Orbitrap data, we observed that approximately one-third of the proteins identified in the LTQ-FT analysis were not detected on the Orbitrap (112 of 320 proteins). Interestingly, most of the proteins that were exclusive to the LTQ-FT analysis (86 hits) are the ones that were validated due to improvements in the Mascot score resulting from MS^3 ^data, and even though most of these 86 hits are present in the Orbitrap data (61 hits), they were discarded due to statistical reasons. On the other hand, the most abundant proteins in the sample had better sequence coverage in the Orbitrap data than in the LTQ-FT data (Figure 3). Discarding single peptide hits with Orbitrap tandem MS data may be overly conservative, since these spectra have very high resolution and low ppm mass errors, making false positives extremely unlikely. If such single peptide hits had been admitted, more than 100 additional proteins could be reported (data not shown). Either way, the presence of a substantial number of single peptide hits suggests that many more proteins are present in this proteome than we report here.

The complete list of proteins identified is summarized in Additional data file 1; this table lists the number of peptides observed for each identified protein, the Mascot score and the MS^3 ^score for each peptide and protein (if available, that is, LTQ-FT data). Protein identification criteria were extremely stringent, requiring fully tryptic peptides with a mass error less than 3 ppm for the LTQ-FT or less than 5 ppm for the Orbitrap. For the FT data, the criteria needed were two matching peptides with a Mascot score of 27 or one matching peptide 'rescued' by an additional MS^3 ^score, adding to a total probability score of at least 54. For the Orbitrap data, the criteria were two matching peptides with minimal score of 21. These criteria ensure an error rate in protein identification of less than 0.1%, so there should be no false positive protein identifications in our data set. In-gel analysis fully covered in-solution identifications. Therefore, all subsequent discussion is based on the in-gel data set.

### Ontology of proteins identified

**Figure 4:**
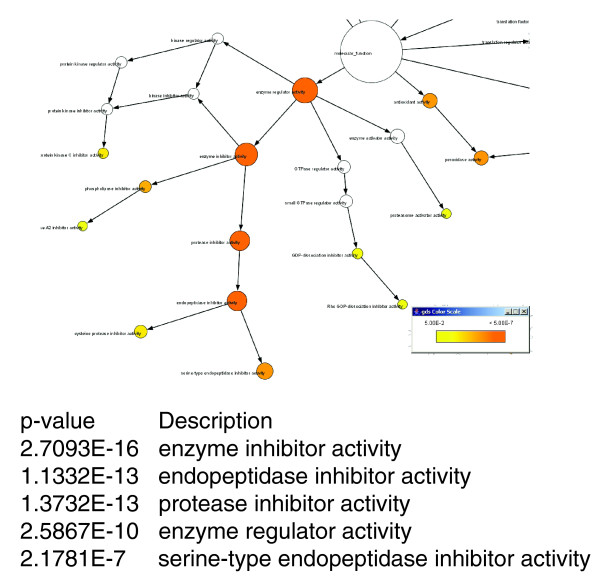
Statistical analysis of GO classification using the BiNGO tool. After identification and merging of the two datasets by the Protein Center tool, a gene list of the 491 identified proteins was generated and submitted to the BiNGO tool. This tool is able to apply statistical analysis to determine over-represented groups present in the sample. The figure shows a partial diagram of the analysis of GO molecular function, zoomed in the protease inhibitors branch. The p values for this group are also indicated.

The 491 proteins identified in the in-gel analysis were functionally classified using the Protein Center Tool (Proxeon Biosystems, Odense, Denmark) and statistical analysis was done using the BiNGO tool [27], based on cellular localization, molecular function and biological process. It should be kept in mind that Gene Ontology (GO) classification and tools that build on those annotations often comprise very broad and overlapping functional categories. Nevertheless, they provide a useful method of initial classification of a large proteome in terms of origin and molecular processes. Figure 4 illustrates an example of group over-representation determined by BiNGO. Tables 1 and 2 list the two main groups of molecular functions identified in this work, and also indicate the biological process that it is involved. Extracellular proteins are indicated by a dagger and proteins already identified in tear samples by an asterisk. Note that the hydrolase GO classification group is very broad, involving several processes, such as signal transduction (phosphatases), energy-driven reactions (ATPases), and glycolysis. We selected from this group proteins that possibly are directly functional in the tear environment, and not only present as a result of cellular degradation in the epithelia, for example. Thus, our 'hydrolase' group listed in Table 1 considers only extracellular proteins, proteins already described as components of the tear fluid, or proteins that participate in biological processes that are known to occur in the fluid that covers the eye. In this way we identified 32 proteins with hydrolase activity, and 32 proteins classified as protease inhibitors, mainly serine protease inhibitors. From these 64 proteins, only 24 proteins had been previously identified as components of tear fluid in other studies.

**Figure 5:**
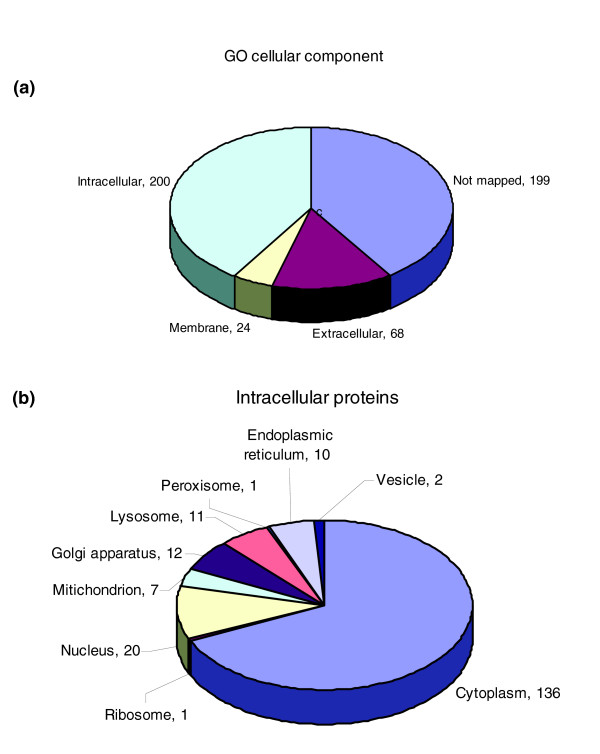
GO classification of tear fluid based on cellular localization. **(a) **Of the 491 proteins identified in the tear fluid, 200 were classified as intracellular proteins, while only 68 were classified as extracellular. As already described in the literature, the presence of intracellular proteins may be a result of cell death in the epithelium in close contact with the eye. **(b) **From the intracellular group, the great majority of proteins belongs to the cytoplasmic region, with some organelles being well represented, such as the lysosome (BiNGO p value of 6.9216E-8, the third highest score after cytoplasmatic and extracellular proteins).

Figure 5a shows the cellular localization pattern of all proteins identified. Approximately 199 proteins were not classified by the ontology database. Interestingly, our data show that 41% (200 proteins) belong to the intracellular compartment, mainly present in cytoplasm (136 proteins), and, to a lesser extent, to compartments such as the nucleus (20 proteins), the Golgi apparatus (12 proteins) and the lysosome (11 proteins). On the other hand, only 68 proteins were classified as extracellular proteins, in addition to 2 that were classified as components of the extracellular matrix. When the not mapped protein group is eliminated from the chart, the intracellular proteins represent approximately 68% of the total identification (Figure 5b).

**Figure 6:**
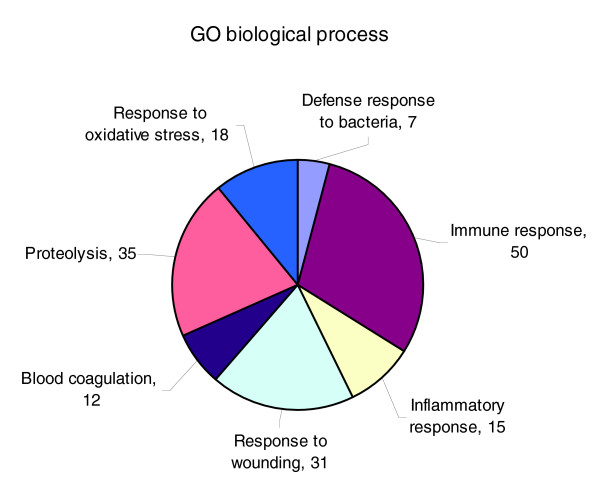
Relevant GO biological processes identified in the tear fluid. In the tear fluid, the most over-represented groups identified according to GO biological process were those involved in defense of the eye environment. These mainly comprised process such as immune response, defense against external biotic agents, response to wounding, and blood coagulation. Interestingly, 18 proteins responsible for response to oxidative stress were identified, only two of them described previously in tear fluid samples.

In addition, the classification of the identified proteins based on biological processes (Figure 6) revealed that at least 37 proteins belong to the immune system, 50 proteins are involved in immune response, such as antibodies and proteins from the complement system, 15 proteins are involved in inflammatory response, and 7 proteins are responsible for defense against pathogens. We also identified 31 proteins that are associated with response to wounding and blood coagulation. Finally, we identified 18 proteins that are involved in the metabolism of reactive oxygen species, such as peroxiredoxins and catalase, which may be functioning in the tear film in the defense against toxic oxygen compounds.

## Discussion

Over the past few decades, less then 80 proteins have been identified in tear fluid in normal or disease states [15]. However, a more comprehensive identification of a larger number of proteins would be desirable to help identify molecular markers of a variety of diseases, such as dry eye syndrome, Sjogren syndrome, complications due to diabetes, conjunctivitis and others [28-30], as well as advance investigation of normal processes of wound healing and immune defense [14,31]. In this study, using a mass spectrometry-based proteomic approach, we identified 491 proteins in tear fluid, using SDS-PAGE fractionation, in-gel trypsin digestion and independent analysis using two different high performance mass spectrometers. We analyzed material from a single, healthy donor as we wanted to characterize the normal tear fluid proteome. The basic composition and make up of such body fluid proteomes are likely to be very similar between healthy subjects, as we have already investigated in more detail in the case of the urinary and saliva proteomes. In these cases, we found that single and pooled samples were identical in terms of their main properties, such as molecular weight distributions and GO classification (Adachi *et al*.: The human urinary proteome contains more than 1,500 proteins, including a large proportion of membrane proteins, *Genome Biology*, in revision; de Souza, Schenk and Mann, unpublished data).

To determine the efficiency of different methods for the characterization of the tear fluid content, we compared an in-gel digestion of tear sample subjected to SDS-PAGE with an in-solution digestion of 1 or 4 μL of tear fluid, all analyzed using the LTQ-FT spectrometer. Our results showed that in-gel digestion identified about five times more proteins than in-solution digestion. This result was unexpected because in-solution digests of protein mixtures, in our experience, can readily identify several hundred proteins in a single analysis [32]. This difference in the number of proteins identified by each method could partly be caused by the high 'dynamic range' of tear fluid, in which 80% to 90% of the protein content is represented by a minor group of proteins [17], which may make the identification of the lower abundant proteins difficult without pre-fractionation of the sample. Although we have no direct evidence, we also speculate that the inefficiency of the in-solution digestion could result from a lack of efficiency of the protocol itself, or from the high number of protease inhibitors and proteases present in the sample.

The large dynamic range of the tear sample could also explain the differences observed in the number of identified proteins between the LTQ-FT and Orbitrap analyses. From the 320 proteins validated in the LTQ-FT data, only two-thirds were also validated in the Oribitrap data. This does not mean that the peptides that lead to a protein identification were not present in the Orbitrap analysis, but it does mean that, due to differences in validation criteria, these hits were not considered statistically significant. Also, as mentioned above, many 'LTQ-FT only' proteins were identified with one peptide in the Orbitrap analysis. Different validation criteria were applied due to the fact that the LTQ-FT instrument performs MS^3 ^analysis while it also performs SIM scans of the precursor ion. We are currently evaluating if one peptide hits in the Orbitrap should also be allowed for protein identification due to the ppm mass accuracy of MS/MS data from this instrument when analysis is performed in the Orbitrap analyzer. We observed that, while the high abundant proteins from the sample were better characterized by the Orbitrap (Figure 3), the proteins identified based on one or two peptides plus the MS^3 ^score still had similar profiles in the Orbitrap. In a situation with a more favorable sample dynamic range, we would expect that proteins identified with one or two peptides in the LTQ-FT would have a larger number of peptides identified in the Orbitrap, due to the higher speed of analysis.

The ontology classification of the identified proteins revealed remarkable characteristics of the tear fluid, so far not described in the literature. Our data show that 200 proteins are primarily classified as intracellular molecules, while only 68 are classified as extracellular proteins. The presence of several intracellular metabolic proteins in tear fluid, such as lactate dehydrogenase, was initially described by van Haeringen and Glasius [33], who also demonstrated that these proteins originate from the cellular shedding of the epithelium that contacts the tear fluid. Most recently, several proteins that perform important functions in tear fluid, such as cathepsins and syaloglycoproteins, were also reported as proteins of epithelial origin [34,35]. In some cases these intracellular proteins may have a functional role in tear fluid, and in other cases they may be present solely as the result of cellular necrosis, but could in principle still be relevant for diagnostic purposes.

We also show that 64 proteins (or approximately 12% of the total number of proteins described) belong to the functional group of hydrolase activity or protease inhibitors. It has been demonstrated that the levels of proteases and proteases inhibitors are in a constant equilibrium in tear fluid [35,36] and that imbalance in these levels may lead to the development of disease states in the eye [31,37]. Our large-scale proteomic investigation greatly extends the number of known proteases and protease inhibitors. These two groups of proteins were the best represented functional group in this study, indicating their importance in tear fluid. Proteins from these groups are associated with defensive mechanisms against pathogens, as well as extracellular matrix remodeling during healing and wounding processes [31,38]. The biological process in which the largest group of proteins is involved is, not surprisingly, the immune defense of the eye. Of the 50 proteins classified as components of the immune defense (immune response, inflammatory response and defense response), 25 were functionally classified as hydrolases or protease inhibitors. The other proteins involved in the immune defense are, mainly, antibodies and proteins from the complement pathway.

We identified 18 proteins that were classified as molecules involved in the response to oxidative stress. It has been demonstrated that the tear fluid possesses anti-oxidative protection against reactive oxygen species (ROS) [39], and decrease in oxidative activity in tear fluid has been associated with several disease states, such as the development of diabetic dry eye disease [40]. The only two proteins related to ROS elimination and already described as components of tear fluid are superoxide dismutase [41,42] and oxyen-regulated protein 1 [13].

## Conclusion

Our proteomic study highlights the importance of the balance of oxidative reactions, as well as the balance of hydrolase activity and protease inhibitors, as we report here 82 proteins involved in these processes (only 26 were described previously). These proteins may play crucial roles in maintaining the eye in a healthy condition. Perturbation of these proteins in the tear fluid may lead to the development of disease states, making them interesting targets for diagnostics and further functional characterization.

## Materials and methods

### Tear sample collection

Samples of closed-eye tear were collected from one of us (GAS) using a 5 μl calibrated glass microcapillary tube (Blauband intraMARK, Brand GMBH, Werthein, Germany) without touching the eye globe or lids, in the course of one week and at different times of the day to avoid diurnal variation [34,35]. One sample typically contained 2 μl. After collection, the tears were centrifuged at 14,000 g for 1 minute at 4°C (Eppendorf model 5417C, Eppendorf, Hamburg, Germany) to remove cellular debris, and stored at -20°C until analysis.

### SDS-PAGE and *in situ *digestion

A tear sample (4 μL) was added to electrophoretic sample buffer (NuPAGE kit, Invitrogen, Karlsruht, Germany) and tear protein content was resolved by SDS-PAGE using a homogeneous 12% gel (NuPAGE gel, Invitrogen) under reducing conditions for 50 minutes with a constant voltage of 200 V. The gel was stained with Coomassie staining kit (NuPAGE, Invitrogen), as instructed by the manufacturer. After staining, two lanes of the gel were combined and then sliced in 13 pieces as indicated in Figure 1. The pieces were then subjected to in-gel reduction, alkylation and tryptic digestion. To reduce disulfide bonds, 100 mM DTT was added to a final concentration of 10 mM in the protein solutions and incubated for 1 h at 56°C in the dark. Free thiol (-SH) groups were subsequently alkylated with iodoacetamide (50 mM final concentration) for 45 minutes at room temperature. The reduced and alkylated protein mixtures were digested with sequence grade-modified trypsin (wt:wt 1:50; Promega, Madison, WI, USA) for 16 h at 37°C in 50 mM NH_4_HCO_3_, pH 8.0. Proteolysis was quenched by acidification of the reaction mixtures with 2% trifluoroacetic acid (Fluka, Buchs, Switzerland). Finally, the resulting peptide mixtures were desalted on RP-C_18 _STAGE tips as described [43] and diluted in 0.1% trifluoroacetic acid for nano-HPLC-MS analysis.

### In-solution digest

Samples of 1 and 4 μl of tear fluid were resuspended in 20 μl of 6 M urea and 2 M thiourea (Invitrogen) and submitted to reduction and alkylation as described above. For enzymatic digestion, Lys-C (wt:wt 1:50; Wako, Japan) was added to the solution for 16 h at room temperature, and the resulting peptides were desalted on RP-C_18 _STAGE tips. The same experiment was repeated using Lys-C for 16 h, followed by trypsin (1:50) for 24 h at room temperature.

### Mass spectrometry

All nano-HPLC-MS^2 ^experiments were performed on an Agilent 1100 nanoflow system connected to a 7-Tesla Finnigan linear quadrupole ion trap-Fourier transform (LTQ-FT) mass spectrometer (ThermoElectron, Bremen, Germany), or connected to a LTQ-Orbitrap mass spectrometer (ThermoElectron), both equipped with a nanoelectrospray ion source (Proxeon Biosystems, Odense, Denmark).

### LTQ-FT

Briefly, for in-gel samples, the mass spectrometer was operated in the data-dependent mode to automatically switch between MS, MS^2^, and MS^3 ^acquisition. Survey full-scan MS spectra (*m*/*z *300 to 1,500) were acquired in the Fourier transform ion cyclotron resonance (FT ICR) with resolution *R *= 25,000 at *m*/*z *400 (after accumulation to a target value of 10,000,000 in the linear ion trap). The three most intense ions were sequentially isolated for accurate mass measurements by an ICR-FT SIM scan with 10 Da mass range, *R *= 50,000 and target accumulation value of 50,000. They were then fragmented in the linear ion trap by collisionally induced dissociation at a target value of 5,000. For MS^3^, up to three ions in each MS^2 ^spectra (the most intense ions with *m*/*z *> 300) were further isolated and fragmented. Former target ions selected for MS^2 ^were dynamically excluded for 30 s. Total cycle time was approximately 3 s. The general mass spectrometric conditions were: spray voltage, 2.4 kV; no sheath and auxiliary gas flow; ion transfer tube temperature, 100°C; collision gas pressure, 1.3 mTorr; and normalized collision energy, 30% for MS^2 ^and 28% for MS^3^. Ion selection thresholds were: 500 counts for MS^2 ^and 50 counts for MS^3^. An activation q-value of 0.25 and an activation time of 30 ms was applied in both MS^2 ^and MS^3 ^fragmentation [20]. However, due to the expected higher complexity of in solution digestion samples, the acquisition method was adjusted to not perform SIM scan or MS^3^, but to sequence the five most intense peaks for obtaining MS^2 ^data.

#### LTQ-Orbitrap

The mass spectrometer was operated in the data-dependent mode to automatically switch between Orbitrap-MS and Orbitrap-MS/MS (MS^2^) acquisition. Survey full scan MS spectra (from *m/z *300 to 1,600) were acquired in the Orbitrap with resolution *R *= 60,000 at *m/z *400 (after accumulation to a target value of 1,000,000 charges in the linear ion trap). The most intense ions (up to five, depending on signal intensity) were sequentially isolated for fragmentation in the linear ion trap using collisionally induced dissociation at a target value of 100,000 charges. The resulting fragment ions were recorded in the Orbitrap with resolution *R *= 15,000 at *m/z *400.

For accurate mass measurements the lock mass option was enabled in both MS and MS/MS mode and the polydimethylcyclosiloxane (PCM) ions generated in the electrospray process from ambient air (protonated (Si(CH3)2O))6; *m/z *= 445.120025) were used for internal recalibration in real time. For single SIM scan injections of the lock mass into the C-trap the lock mass 'ion gain' was set at 10% of the target value of the full mass spectrum. When calibrating in MS/MS mode the ion at *m/z *429.088735 (PCM with neutral methane loss) was used instead for recalibration [24].

Target ions already selected for MS/MS were dynamically excluded for 30 s. General mass spectrometric conditions were: electrospray voltage, 2.4 kV; no sheath and auxiliary gas flow; ion transfer tube temperature, 125°C; collision gas pressure, 1.3 mTorr; normalized collision energy, 32% for MS^2^. Ion selection threshold was 500 counts for MS^2^. An activation q-value of 0.25 and activation time of 30 ms was applied for MS^2 ^acquisitions.

### Data analysis

Stringent criteria were applied for protein identification, which was performed by searching the data against the International Protein Index database (IPI_human) by MASCOT (Matrix Science) and MSQuant (an in-house developed, open source software program). These criteria comprised: for LTQ-FT-ICR data, a mass accuracy within 3 ppm (in-gel digestion; average absolute peptide mass accuracy was 1.03 ppm) or 25 ppm (in-solution digestion; average absolute accuracy was 8.3 ppm); for LTQ-Orbitrap data, a mass accuracy of 5 ppm (average absolute accuracy of 1.01 ppm); at least two, fully tryptic, matching peptides per protein with a Mascot score for individual peptides (MS^2^) better than 27 (p ≤ 0.01), or one peptide with MS^2 ^+ MS^3 ^score better that 54 (p ≤ 0.0001), when MS^3 ^was performed. For in-solution digestion, proteins were considered identified if they had at least two peptides with score higher than 35. For Orbitrap data, the criteria were a mass accuracy within 3 ppm (average absolute peptide mass accuracy was 1.22 ppm) and at least 2 fully tryptic peptides per protein with a Mascot score better then 21 (p ≤ 0.01) for individual peptides (MS^2^). Differences in mass accuracy between in-solution samples (more complex compared to in-gel samples due to lack of pre-fractionation) was observed because the measurement of ion masses was not performed with the SIM method, leading to higher sequencing speed of the method at the cost of lower mass accuracy.

Experiments with a reversed database were performed as described in [44]. The number of statistically significant peptides identified in the IPI database was 1,935, while the reverse database identified 12 peptides with statistical significance (0.6%) for the LTQ-FT data. However, these 12 peptides were not sufficient to identify a single protein (that is, none of the proteins had at least 2 peptides with score higher than 27 or one peptide with MS^3 ^score higher than 54). The Orbitrap data included no peptides within statistical significance in the reverse database. Identified proteins were combined in a larger data set and initial GO characterization was done using the Protein Center tool (v0.62, Proxeon Biosystems).

### Data

Our data are freely available at the proteome database of the department of proteomics and signal transduction of the Max-Planck-Institut for Biochemistry [45].

## Additional data files

The following additional data are available with the online version of this paper. Additional data file 1 lists all peptides and protein hits obtained in both LTQ-FT and LTQ-Orbitrap data.

## Supplementary Material

Additional data file 1 All peptides and protein hits obtained in both LTQ-FT and LTQ-Orbitrap data. Click here for file

## Figures and Tables

**Table 1:** Hydrolases identified in tear fluid

Hydrolase activity	Gene symbol	Biological process
Leukotriene A-4 hydrolase	LTA4H	1,2
Matrix metalloproteinase 8*	MMP8	1,3
Matrix metalloproteinase 9*	MMP9	1,3
Myeloblastin^†^	PRTN3	3
Apolipoprotein B	APOBEC3C	4
Azurocidin^†^	AZU1	4
Dipeptidylpeptidase IV	DPP4	4
Leukocyte elastase*	ELA2	4
Haptoglobin*	HP	4
Lactotransferrin*	LTF	4,5
Lysozyme C*	LYZ	4
Eosinophil cationic protein*	RNASE3	4,9
Adipocyte-derived leucine aminopeptidase^†^	ARTS-1	5
Zinc-alpha-2-glycoprotein*	AZGP1	5
Complement factor B^†^	BF	5
Ubiquitin thiolesterase protein^†^	OTUB1	5
Palmitoyl-protein thioesterase 1	PPT1	6
Plasminogen*	PLG	7
Aminopeptidase N	ANPEP	8
Acid phosphatase, prostate^†^	ACPP	10
Chitinase 3-like protein 2^†^	CHI3L2	11
Cytosolic nonspecific dipeptidase^†^	CNDP2	1
Cathepsin B*	CTSB	1
Cathepsin D	CTSD	1
Cathepsin G	CTSG	1
Cathepsin Z	CTSZ	1
Prostasin^†^	PRSS8	1
Aminopeptidase B^†^	RNPEP	1
Tissue alpha-L-fucosidase	FUCA1	12
Beta-mannosidase*	MANBA	13
Alpha-N-acetylglucosaminidase*	NAGLU	12
Neuraminidase	NEU1	12

*The protein has already described in the tear fluid. ^†^The protein is classified as an extracellular protein. 1, Proteolysis; 2, inflammatory response; 3, extracellular matrix degradation; 4, defense response; 5, immune response; 6, visual perception; 7, blood coagulation; 8, angiogenesis; 9, nucleotide metabolism; 10, regulation of cell proliferation; 11, chitin catabolism; 12, carbohydrate metabolism; 13, protein modification; 14, central nervous system development; 15, signal transduction.

**Table 2:** Protease inhibitors identified in the tear fluid

Protease inhibitor	Gene symbol	Biological process
Alpha-2-macroglobulin*	A2M	
Alpha-2-HS-glycoprotein*	AHSG	2
Alpha-1-microglobulin^†^	AMBP	2
Annexin 5	ANXA5	7
Complement C3*	C3	2,5
Complement C4^†^	C4A	2,5
Cystatin B	CSTB	
Cystatin SN*	CST1	
Cystatin SA*	CST2	
Cystatin C*	CST3	
Cystatin S*	CST4	
Inter-alpha-trypsin inhibitor heavy chain H1*	ITIH1	5
Inter-alpha-trypsin inhibitor heavy chain H2^†^	ITIH2	5
Inter-alpha-trypsin inhibitor heavy chain H4^†^	ITIH4	5
Lipocalin 1*	LCN1	5
Similar to Lipocalin 1		
Lipocalin 2	LCN2	
Latexin^†^	LXN	
Prosaposin^†^	PSAP	
Alpha-1-antitrypsin*	SERPINA1	5
Alpha-1-antichymotrypsin*	SERPINA3	2,5
Leukocyte elastase inhibitor*	SERPINB1	5
Plasminogen activator inhibitor-2^†^	SERPINB2	7
Maspin^†^	SERPINB5	
Placental thrombin inhibitor^†^	SERPINB6	
Antithrombin-III^†^	SERPINC1	7
Pigment epithelium-derived factor*	SERPINF1	8
Plasma protease C1 inhibitor^†^	SERPING1	5,7
Neuroserpin	SERPINI1	14
Stratifin^†^	SFN	15
Thrombospondin-1^†^	THBS1	7
Tissue inhibitor of metalloproteinase 1*	TIMP1	10

*The protein has already described in the tear fluid. ^†^The protein is classified as an extracellular protein. 1, Proteolysis; 2, inflammatory response; 3, extracellular matrix degradation; 4, defense response; 5, immune response; 6, visual perception; 7, blood coagulation; 8, angiogenesis; 9, nucleotide metabolism; 10, regulation of cell proliferation; 11, chitin catabolism; 12, carbohydrate metabolism; 13, protein modification; 14, central nervous system development; 15, signal transduction.
